# Global research trends in the intestinal microflora and depression: bibliometrics and visual analysis

**DOI:** 10.3389/fcimb.2025.1507667

**Published:** 2025-02-25

**Authors:** Qian Xu, Qingwei Xiang, Zihu Tan, Qiong Yang

**Affiliations:** ^1^ School of Clinical Traditional Chinese Medicine, Hubei University of Chinese Medicine, Wuhan, China; ^2^ Hubei Provincial Hospital of Traditional Chinese Medicine, Wuhan, China

**Keywords:** depression, intestinal flora, research hotspot, research trend, bibliometric analysis

## Abstract

**Background:**

In recent years, the relationship between gut microbiota and human health has garnered significant attention. Notably, the potential connection between gut microbiota and mental health issues, such as depression and anxiety, has emerged as a new focal point for research. While some studies suggest a possible link between these factors, the field remains in its early stages of development, and there are notable methodological and sample size limitations.

**Purpose:**

This study aims to systematically summarize the knowledge systems, research hotspots, and development trends related to intestinal microflora within the context of depression research.

**Methods:**

This study conducted a search for publications related to intestinal microflora and depression in the Web of Science Core Collection (WOSCC) prior to August 6, 2024. The selected literature was subsequently analyzed using VOSviewer (v.1.6.20), SCImago Graphica (v.1.0.39), and CiteSpace (v.6.3.1).

**Results:**

The study encompassed a total of 1,046 publications, demonstrating a consistent increase in annual publication volume. The primary research countries identified are China and the United States, with notable contributions from institutions such as the University of California and University College Cork, among others. Keywords analysis highlighted high-frequency terms including “gut microbiota,” “depression,” and “anxiety,” and revealed 10 keyword clusters along with 20 strongest citation bursts keywords. The focus of research has shifted from compositional analysis of gut microbiota to its role in the pathogenesis of depression.

**Conclusions:**

Research on gut microbiota and depression is growing, but there is still a need for greater collaboration between authors and institutions across regions, more ongoing interaction and communication to further explore the mechanisms of action of gut microbiota, to develop microbiota-based interventions, and to facilitate translation of research findings into clinical practice.

## Introduction

Depression, characterized by a significant and persistent low mood, is a mental disorder that presents a substantial health challenge worldwide. It is marked by a “high incidence, high disability, high recurrence rate, and high mortality rate.” Over the past 30 years, the number of global cases has increased by nearly 50% (Liu et al., 2020), with a recurrence rate of 75% to 90% following the initial depressive episode (Solomon et al., 2000). In China, the prevalence of depression is approximately 7.4% and continues to rise annually (Lu et al., 2021). According to the World Health Organization (WHO), depression is a leading cause of mental and physical disability and a significant contributor to the global burden of disease (Ara, 2022). Current preventive and treatment measures for depression are insufficient; therefore, understanding its etiology and disease progression is crucial for developing early diagnostic techniques, advanced treatment strategies, and comprehensive rehabilitation plans.

The gut microbiota is an ecosystem composed of trillions of bacteria, viruses, Archaea, and fungi that plays a crucial role in human health (Mulder et al., 2024). The two-way communication pathway between these microorganisms and the host’s central nervous system is referred to as the gut-brain axis, which influences cognitive function and mood through neural, metabolic, hormonal, and immune-mediated mechanisms (Simpson et al., 2021). A growing body of research indicates that alterations in the composition and function of the gut microbiota are linked to the onset and progression of depression, primarily through modulation of the gut-brain axis (Liu et al., 2023a; Wang et al., 2024). In recent years, significant progress has been made in the study of the gut microbiome and depression, both nationally and internationally, focusing mainly on the composition of the gut microbiome, the function of the gut-brain axis, and the pathogenesis of depression. However, there are relatively few reports on bibliometric analysis and visualization research in this field. Therefore, it is of great significance to conduct a visual analysis of the status quo, hot spots, and frontiers of research on gut microbiota and depression, which not only helps to comprehensively sort out the research progress in this field, but also provides a reference for future research directions and promotes the in-depth development of research in this field.

In the realm of Informatics, bibliometrics provides a detailed analysis of scholarly literature through both quantitative and qualitative approaches. This methodological rigor facilitates a macro-level understanding of the trajectory and progress within specific research domains (Wei et al., 2022). Our study explores the correlation between depression and the gut microbiota, utilizing the analytical capabilities of Citespace and Vosviewer to visually represent data sourced from the Web of Science database. The objective is to identify and emphasize trends and focal points in global research, thereby establishing a substantial foundation for future investigations into depression.

## Materials and methods

### Literature sources and search strategies

The core collection of Web of Science was utilized as the primary source of literature, and advanced search methodologies were employed. To enhance the reliability and representativeness of the literature, we utilized the MeSH terms provided by PubMed as keywords for the search. The search formula was as follows: ((((((((TS=(“Intestinal flora”)) OR TS=(“Gastrointestinal Microbiome”)) OR TS=(“Gastrointestinal Microbiome*”)) OR TS=(“Microbiome, Gastrointestinal”)) OR TS=(“Gastrointestinal Microbial Community”)) OR TS=(“Gastrointestinal Microbial Communities”)) OR TS=(“Gut Microbiome*”)) OR TS=(“Gut Microflora”)) OR TS=(“Gastrointestinal Microflora”) AND ((((((TS=(Depression)) OR TS=(“Depressive Symptom*”)) OR TS=(“EmotionalDepression”)) OR TS=(“Depressive Disorder*”)) OR TS=(“Neurosis, Depressive”)) ORTS=(“Depressive Neuroses”)) ORTS=(“Depression, Emotional”). The search period spanned the period from the inception of the database to August 6 2024.

### Inclusion and exclusion criteria

The inclusion criteria for this study encompassed both original articles and review papers that specifically addressed the relationship between depression and gut microbiota. The exclusion criteria eliminated irrelevant literature, duplicate publications, studies that lacked full access to information or were incomplete, as well as editorial material, conference abstracts, book chapters, withdrawn documents, and any other unrelated literature. Literature screening, data extraction, and cross-examination were conducted independently by two researchers, with any objections submitted to the research team for evaluation.

### Data analysis and visualization

In this study, four scientific tools were employed for bibliometric and visualization analysis. Microsoft Office Excel 2022 was used to organize the publications and analyze their basic information.VOSviewer (v.1.6.20) is a widely used bibliometric visualization software for author co-author analysis, journal analysis, and keyword co-occurrence analysis. CiteSpace (v.6.3.1) is another mainstream bibliometric tool, mainly used for visualizing co-citation networks and identifying institutions, key references, strongest citations, keyword emergence, clustering, and timeline plots. Additionally, CiteSpace has created dual map overlays of journals. SCImago Graphica (v.1.0.39) software maps data to visual attributes through the graphic grammar engine, thus realizing data visualization, primarily for country/region analysis.

The selected documents were imported into CiteSpace (v.6.3.1) for analysis. Upon import, the software parameters were configured with the time slicing database set to 2024 and a default time interval of one year. Node types such as institutions, authors, and keywords were selected. The pruning parameter was set to the Pathfinder method, and the data were subsequently processed to identify hot spots and boundaries in the field. Additionally, VOSviewer (v.1.6.20) and SCImago Graphica (v.1.0.39) were employed to analyze countries, journals, authors, and keywords, among other aspects. The main parameters were configured as follows: Size: frequency; Color: clusters; Label: selected according to the content of the analysis, utilizing the same color as labels; Layout: Circular; Edges: matching the color of the labels. In the visualization network diagram, nodes represent various parameters such as countries, institutions, and keywords, and the size of the nodes indicates their relative importance. The differently colored lines connecting each node depict their interactions, with the thickness of the lines reflecting the strength of the correlation between the nodes;​thicker lines indicate a stronger connection. By combining these complementary indicators and analytical tools, we aim to identify key trends, influential authors, institutions, and promising research directions, and to generate a comprehensive and nuanced understanding of the research landscape across bibliometric dimensions such as productivity, impact, collaboration, and thematic focus. Ultimately, these analyses provide a valuable reference for the research field of gut microbiota in depression.

## Results

### Literature search results

**Figure 1:**
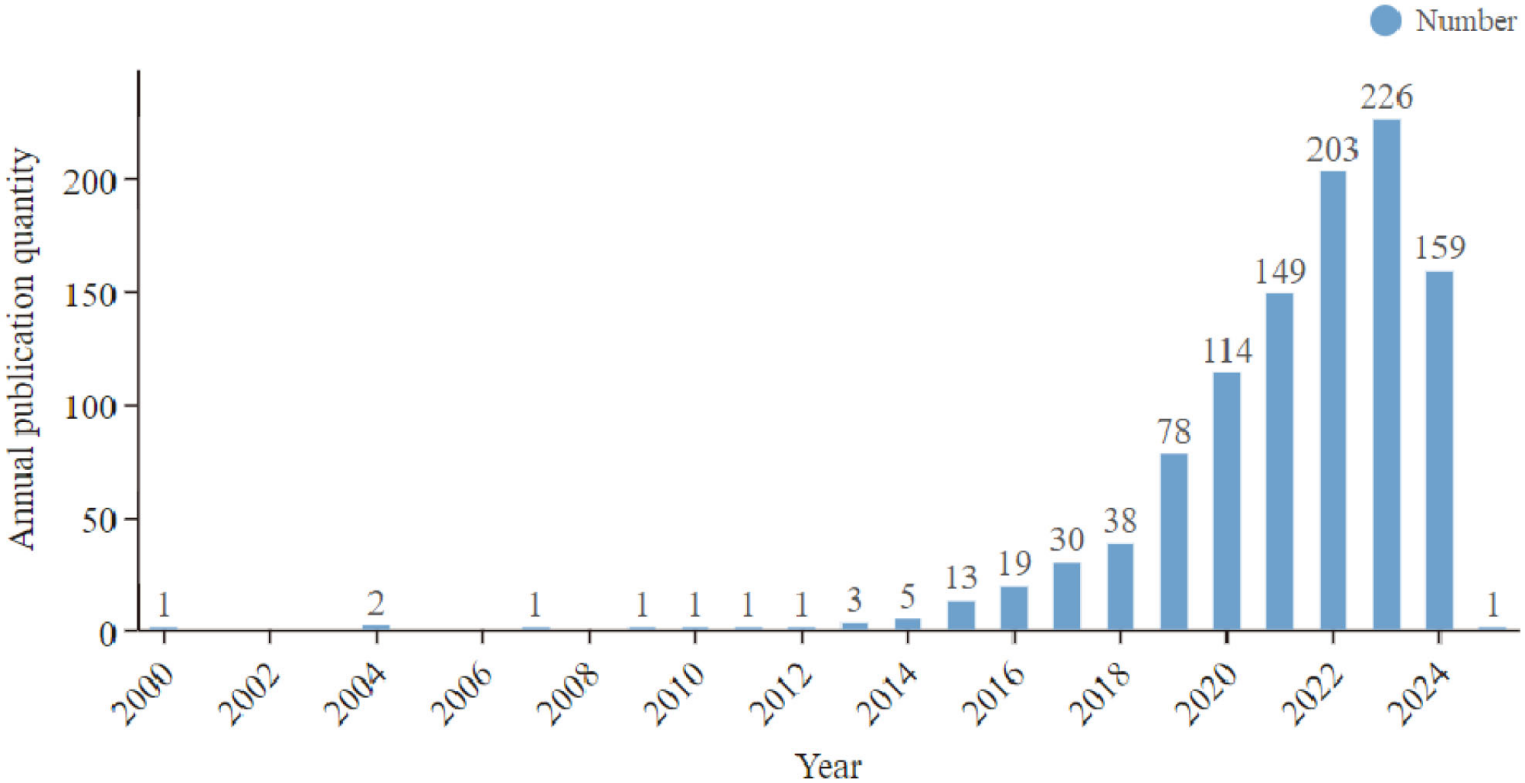
Depression and intestinal flora related publication volume overview. The annual publication number during 2000 and 2024.

A total of 1,046 publications were ultimately included in this analysis. The Web of Science database recorded the first study on the relationship between depression and gut microbiota in 2000. Since that year, the annual publication volume in this research area has exhibited an overall growth trend. From 2000 to 2014, the number of publications remained low and stable. However, after 2015, there was a significant increase, culminating in a peak of 226 articles published in 2023. The rapid increase in the number of papers published after 2015 is closely linked to major advances in microbiome research. For example, the Human Microbiome Project (HMP), launched in 2012, and the European MetaHIT project have provided a large amount of high-quality data and tools for studying the gut microbiome. Around 2015, several high-impact studies revealed a strong link between the gut microbiome and brain function, a concept known as the gut-brain axis. Top journals such as *Nature* and *Science* have published several reviews and original studies on the relationship between the gut microbiota and neuropsychiatric disorders, further boosting the field. It is important to note that the publication count for 2024 is based solely on data collected up to August 6 and may not accurately represent the total number of publications for the entire year (**Figure 1**).

### Countries/regions distribution and cooperation

**Figure 2:**
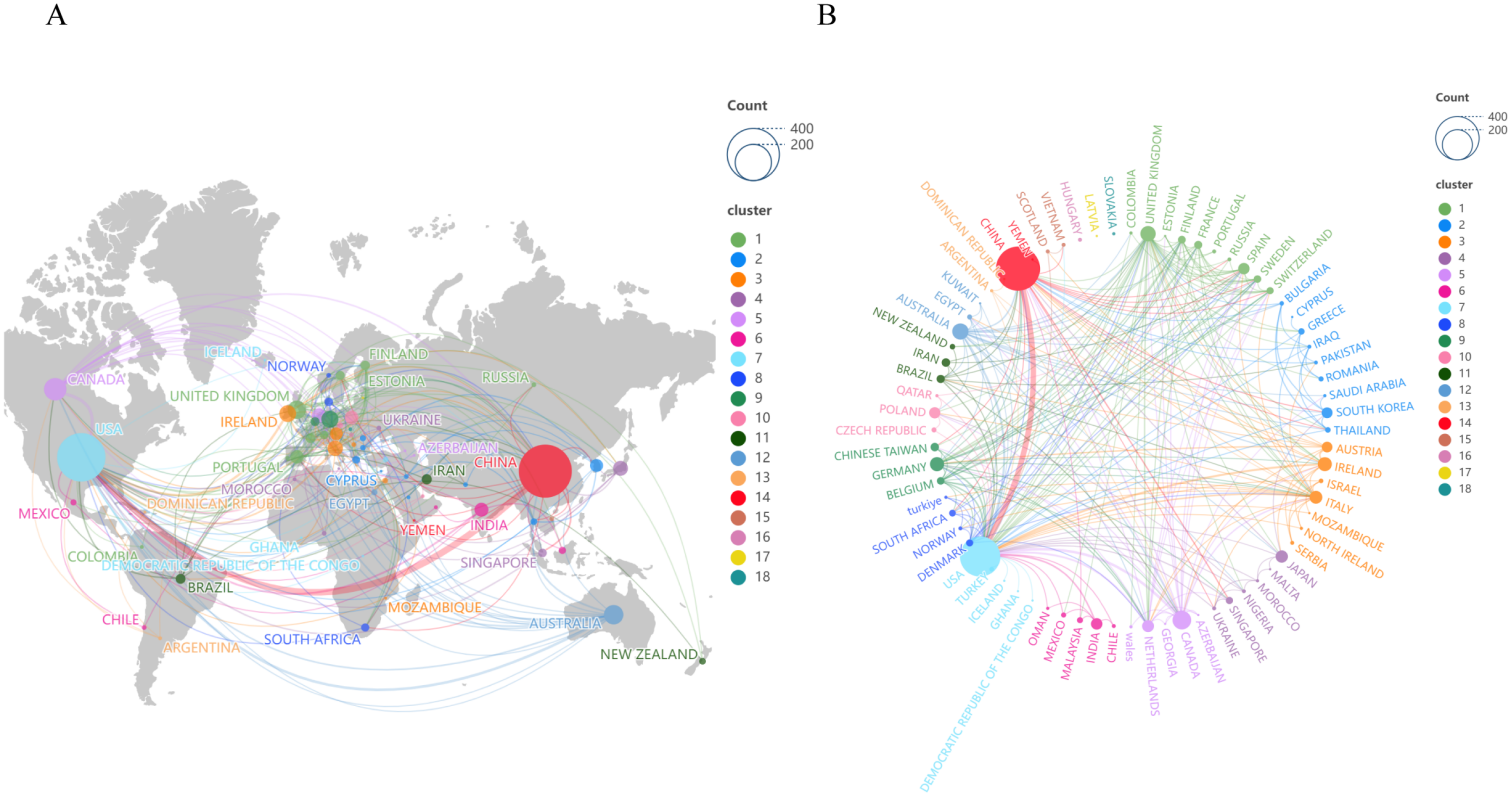
The involvement of various countries/regions in depression and intestinal flora research. **(A)** World map of depression and gut flora research. Countries/regions are represented by circles, while lines illustrate their collaborations. The weight corresponds to the publication count, line thickness denotes the strength of collaboration, and distinct colors highlight clusters. **(B)** Network diagram assessing the global collaboration.

**Figure 2** illustrated the co-occurrence map of countries and regions involved in the study of depression and gut microbiota. Since the inception of the database, a total of 72 countries and regions have contributed to this field of research. Governments and research institutions support research in this area through research funding, international cooperation programs and policy support. **Table 1** listed the top 10 countries and regions with the highest publication outputs, with China leading by publishing 381 papers, which accounted for 36.4% of the total. The United States, Canada, and Australia followed closely behind. Furthermore, the connecting lines in the figure represented the collaborative ties among countries and regions globally, forming 18 distinct clusters.

**Table 1:** Top 10 countries/regions in terms of publications.

Rank	Country/Region	Publications	Citations	Centrality
1	CHINA	381	9158	0.09
2	USA	317	16995	0.21
3	CANADA	68	2782	0.27
4	AUSTRALIA	51	3203	0.02
5	ENGLAND	46	1136	0.5
6	GERMANY	39	3268	0.3
7	IRELAND	38	4247	0.13
8	ITALY	32	1020	0.3
9	JAPAN	30	819	0.11
10	NETHERLANDS	28	2294	0.75

### Analysis of cooperation among document publishing institutions

**Figure 3:**
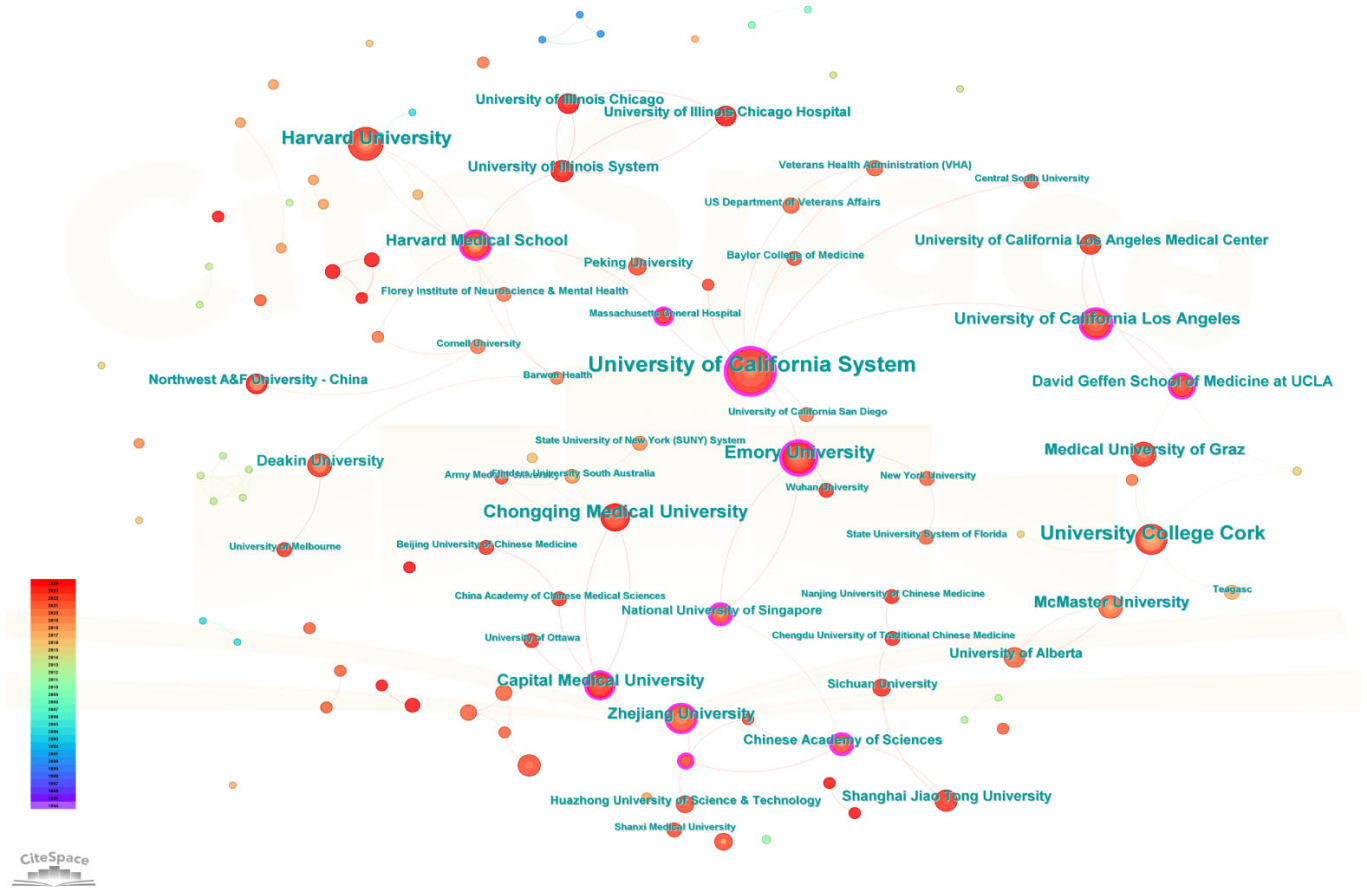
Visualization of institutions related to the research on depression and intestinal flora. Co-occurrence network view of institutions.

**Figure 3** offered a graphical overview of institutions that were engaged in research on the gut microbiota in relation to depression. **Table 2** specified the top 10 academic units within these institutions, with the University of California, University College Cork, and Harvard University leading the list due to their substantial publication output. Notably, the University of California, which had the highest centrality score of 0.35, was identified as the most influential institution in this area of research. Its leading position is due to its strong scientific research infrastructure, interdisciplinary research capabilities, and abundant scientific research funding support. In China, Chongqing Medical University, Capital Medical University, and Zhejiang University had also demonstrated notable research strength, positioning themselves among the top 10 institutions based on the number of publications.

**Table 2:** Top 10 institutions in terms of publications.

Rank	Institution	Publications	Centrality
1	University of California System	43	0.35
2	University College Cork	28	0.08
3	Harvard University	23	0
4	Emory University	21	0.26
5	Chongqing Medical University	19	0.07
6	University of California Los Angeles	16	0.14
7	Capital Medical University	16	0.13
8	Harvard Medical School	14	0.19
9	Zhejiang University	14	0.14
10	Medical University of Graz	14	0

### Author analysis

**Figure 4:**
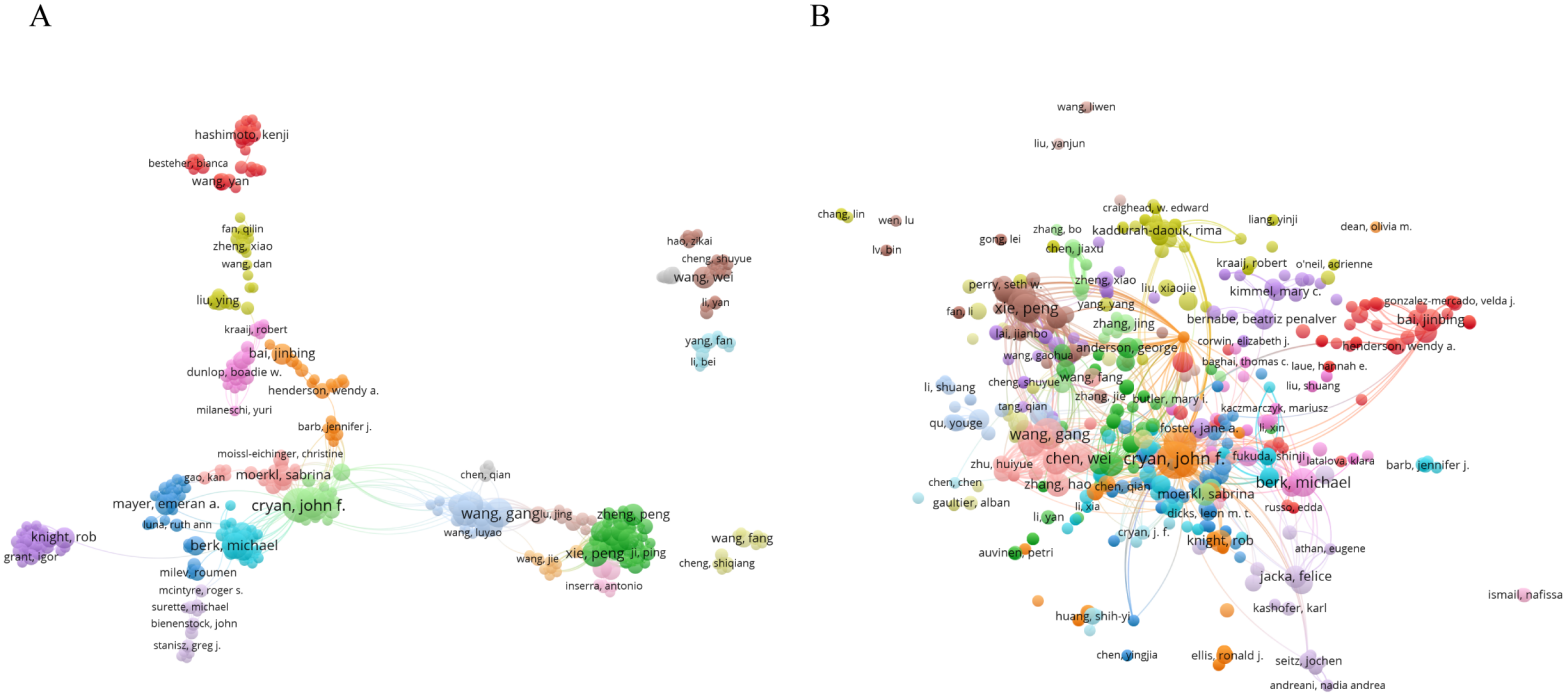
Visual analysis of publishing authors **(A)** The cooperation network of co-occurring authors visualized using VOSviewer. **(B)** Co-cited authors.

**Figure 4** illustrated the significant contribution of 6,153 authors to the research on gut microbiota in relation to depression. **Table 3** highlighted the top 10 most prolific and influential authors in this field. Notably, Cryan John F emerged as the author with the highest number of publications and citations. These distinguished authors had naturally formed various clusters, indicating the establishment of strong collaborative networks among them.

**Table 3:** Top 10 authors by number of publications and citations.

Rank	Author	Number of publications	Author	Citations
1	John F. Cryan	19	John F. Cryan	2491
2	Timothy G. Dinan	15	Timothy G. Dinan	2480
3	Wang Gang	13	Gerard Clarke	2094
4	Chen Wei	12	Emeran A. Mayer	1312
5	Xie Peng	11	Rob Knight	962
6	Michael Berk	11	Michael Berk	740
7	Tian Peijun	10	Wang Yan	564
8	Zhao Jianxin	10	Xie Peng	498
9	Gerard Clarke	9	Zheng Peng	469
10	Zheng Peng	8	Felice Jacka	408

### Analysis of journals and cited journals

**Figure 5:**
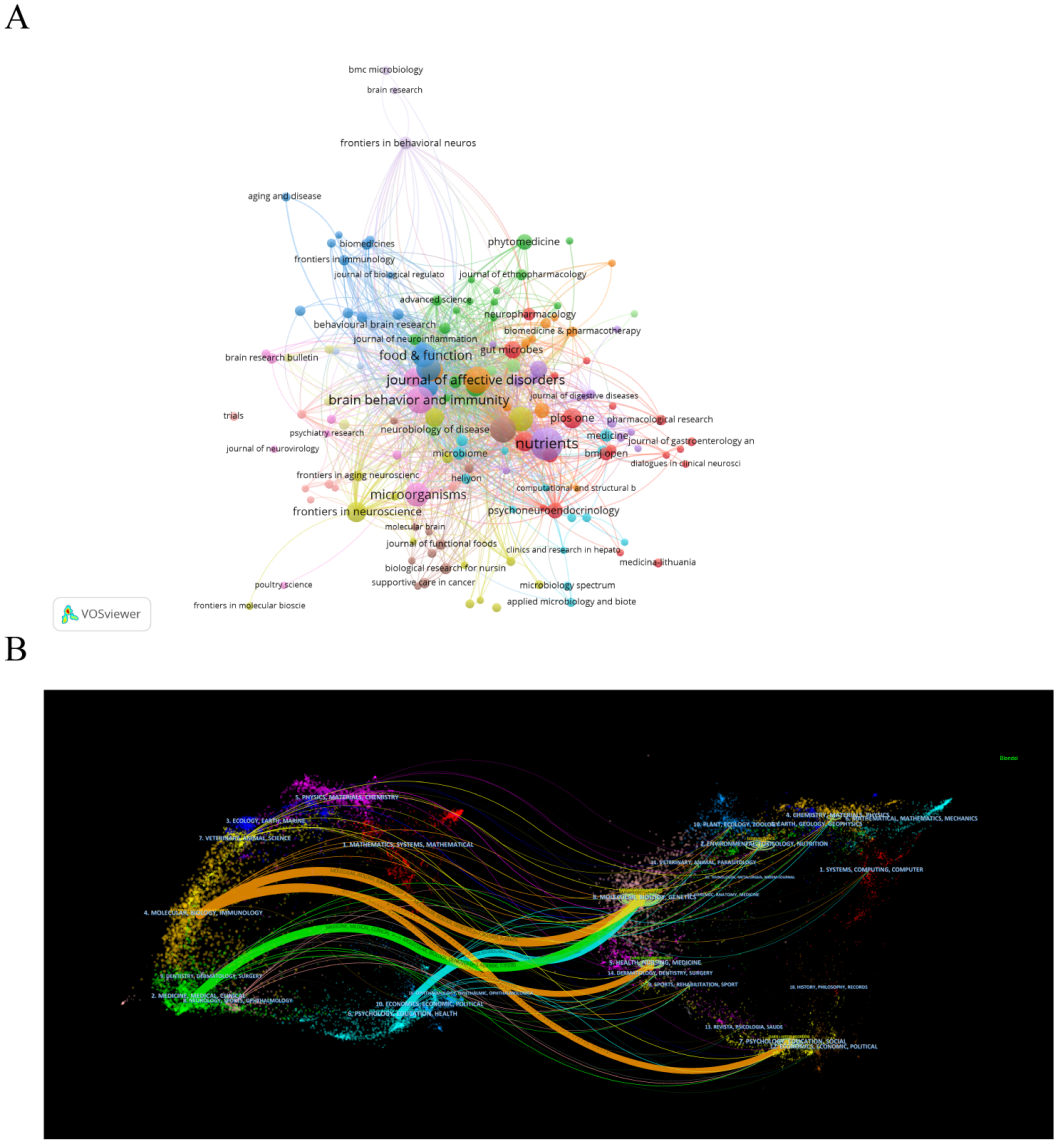
Journals visualization analysis related to depression and intestinal flora. **(A)** Co-occurrence network view of journals. The nodes indicate the number of citations, while the links represent the intensity of cooperation. **(B)** Dual-map overlay of journals. The dual-map overlay of journals displays the associations between publications and citations, with dots representing citing journals in the left and cited journals in the right, so that the citation relationships are depicted as colored lines from the left to the right.

Research on depression and gut microbiota has been published in 426 scientific journals, as shown in **Figure 5A**. **Table 4** highlights the top 10 journals with the highest publication volumes and citation rates. Notably, *Nutrients, Journal of Affective Disorders, Brain, Brain Behavior and Immunity* emerged as the leading journals, contributing 39, 39, and 27 articles, respectively. These journals have significantly promoted the dissemination of research on the relationship between gut microbiota and depression by publishing high quality research papers and reviews. In addition, journals such as *Molecular Psychiatry, Brain Behavior and Immunity, Oikos, and Nutrients* had higher citation rates, reflecting their authority in the fields of mental health and immunology. The dual map overlay analysis shown in **Figure 5B** revealed four main citation pathways. The citing journals were mainly categorized under “Molecular/Biology/Immunology” and “Medicine/Medical/Clinical” while the cited journals were mainly found in the fields of “Molecular/Biology/Genetics”, “Health/Nursing/Medicine” and “Psychology/Education/Social”. This phenomenon highlights the significant interdisciplinary nature of research on depression and gut microbiota.

**Table 4:** Top 10 journals in terms of publications.

Rank	Journal	Number of publications	JCI	IF	Citation	H-index
1	Nutrients	39	Q1	4.8	1390	75
2	Brain Behavior and Immunity	27	Q1	8.8	1685	127
3	Journal of Affective Disorders	27	Q1	4.9	535	165
4	Frontiers in Psychiatry	25	Q2	3.2	1082	52
5	International Journal of Molecular Sciences	22	Q2	4.9	646	114
6	Frontiers in Microbiology	22	Q2	4	142	88
7	Microorganisms	21	Q2	4.1	211	
8	Food & Function	21	Q1	5.1	470	53
9	Scientific Reports	17	Q1	3.8	351	149
10	Frontiers in Neuroscience	15	Q2	3.2	176	71

### Analysis of references

**Figure 6:**
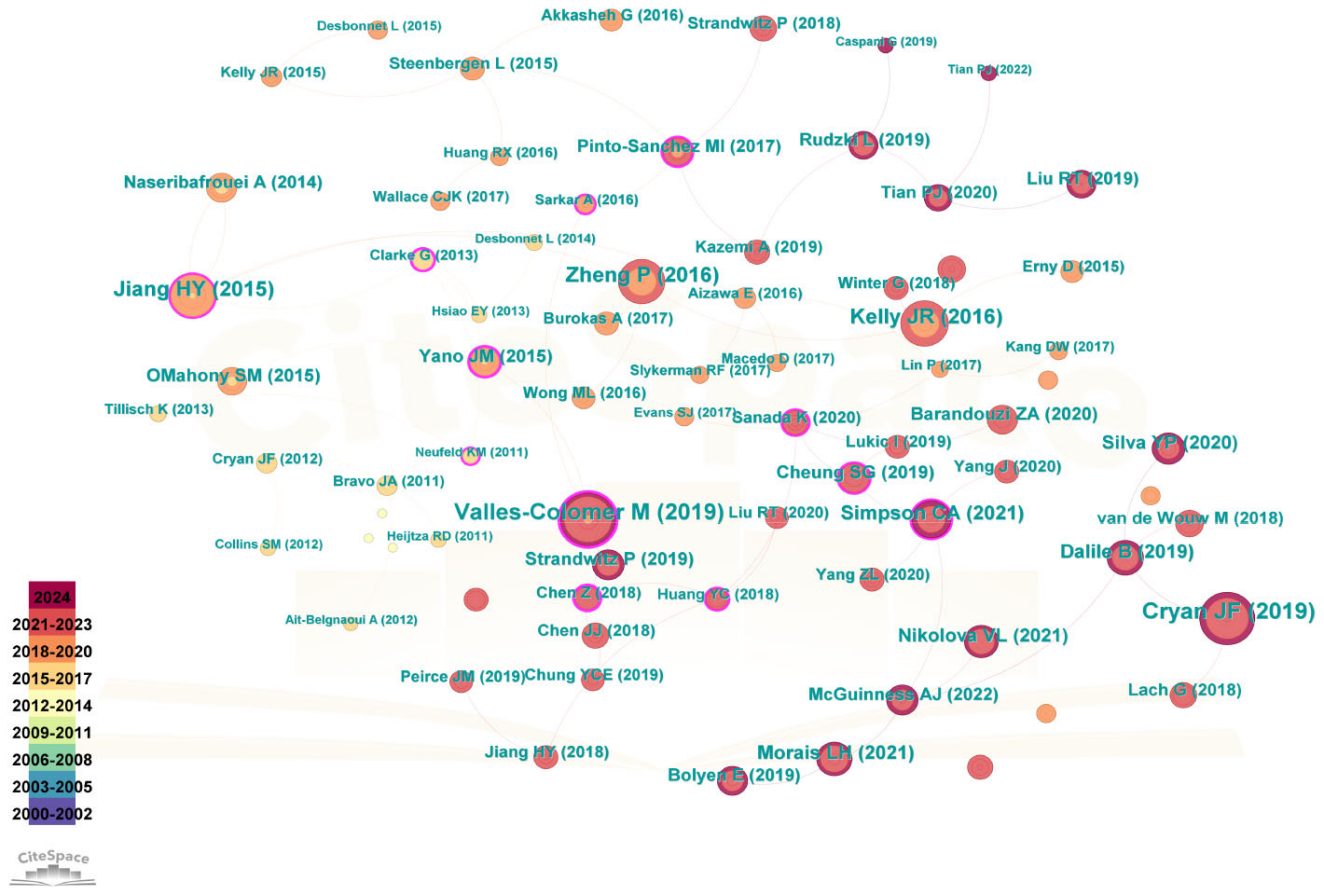
Co-cited references concerning depression and intestinal flora. The visual network of references to work on depression and intestinal flora.

Highly cited literature has frequently served as a vital knowledge base for research and development in this field. A total of 1,046 publications were retrieved, leading to the establishment of 146 nodes and 251 connections, with a cumulative citation frequency of 59,382, as illustrated in **Figure 6**. Among these, the top-cited references include “The Microbiota-Gut-Brain Axis” and “The Neuroactive Potential of the Human Gut Microbiota in Quality of Life and Depression.” These publications have been instrumental in shaping the understanding of how microbial balance in the gut can influence mental health, suggesting potential avenues for therapeutic intervention and marking a significant stride in the field of psychobiotics and mental health research.

### Keywords analysis

**Figure 7:**
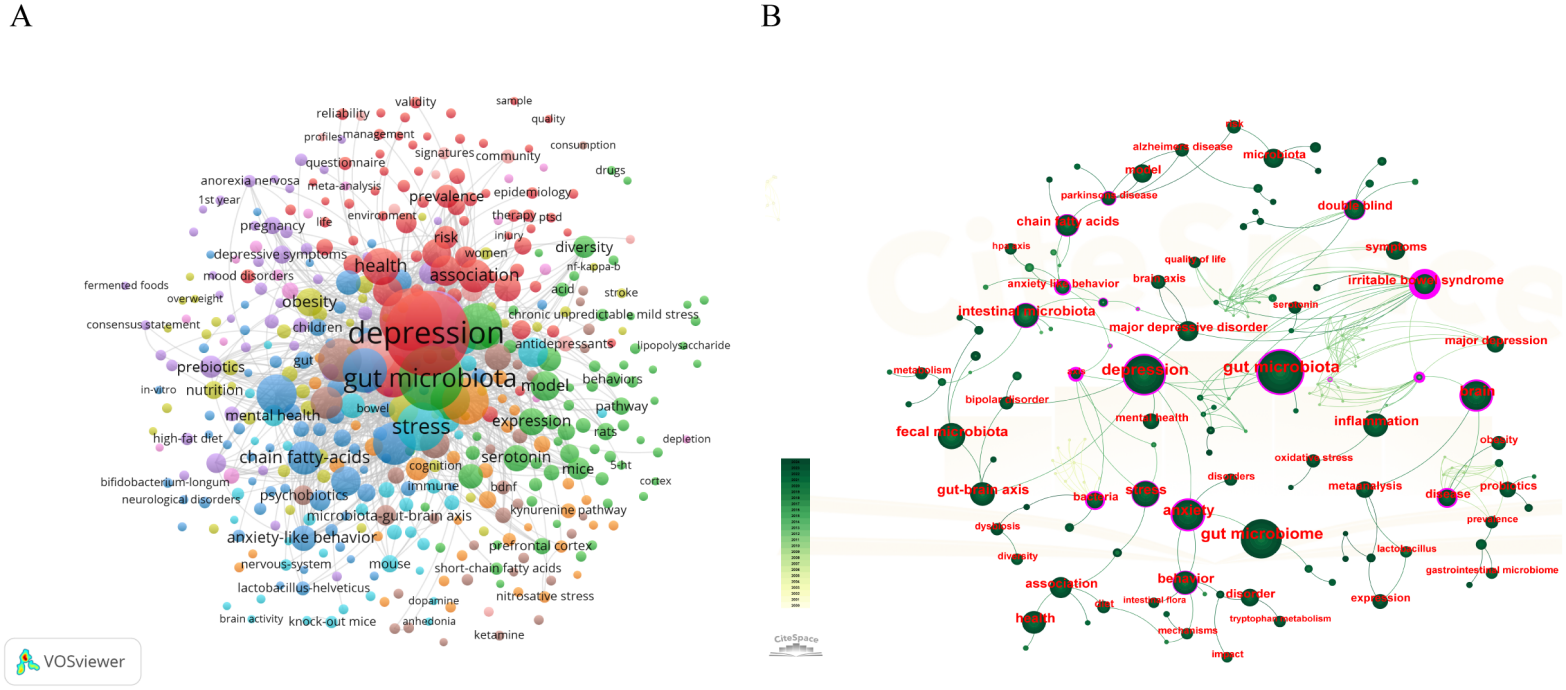
The representation of keyword mapping focusing on depression and intestinal flora. **(A)** Co-occurrence network view of keywords. Node size reflects the frequency of occurrence. **(B)** The visual network of keywords to work on depression and intestinal flora.

Keywords served as concise indicators of a research paper’s primary focus, encapsulating the essence of the research questions and key findings. They provided a snapshot of the study’s subject matter and its contributions to the field. By examining these keywords, the research hotspots, developmental trends, and areas of concentrated interest within the academic community at that time could be delineated. **Figure 7** visually represented the high-frequency keywords, facilitating the identification of prominent areas of interest and prevailing trends in the study of depression and gut microbiota. **Table 5** further categorized and listed the top 15 keywords by their frequency of occurrence. Notably, terms such as ‘gut microbiota,’ ‘depression,’ ‘anxiety,’ and ‘brain’ were prominently featured, underscoring their significance in the discourse.

**Table 5:** Top 15 keywords with the highest frequency of occurrence.

Rank	Publications	Centrality	Year	Keywords
1	318	0.26	2015	gut microbiota
2	283	0.04	2016	gut microbiome
3	268	0.3	2015	depression
4	171	0.22	2016	anxiety
5	144	0.34	2013	brain
6	134	0.05	2016	fecal microbiota
7	122	0.1	2014	stress
8	116	0.15	2014	intestinal microbiota
9	114	0.1	2016	gut-brain axis
10	105	0.15	2017	behavior
11	102	0.04	2018	inflammation
12	98	0.02	2017	health
13	81	0.19	2016	chain fatty acids
14	80	1.02	2010	irritable bowel syndrome
15	78	0.08	2018	association

**Figure 8:**
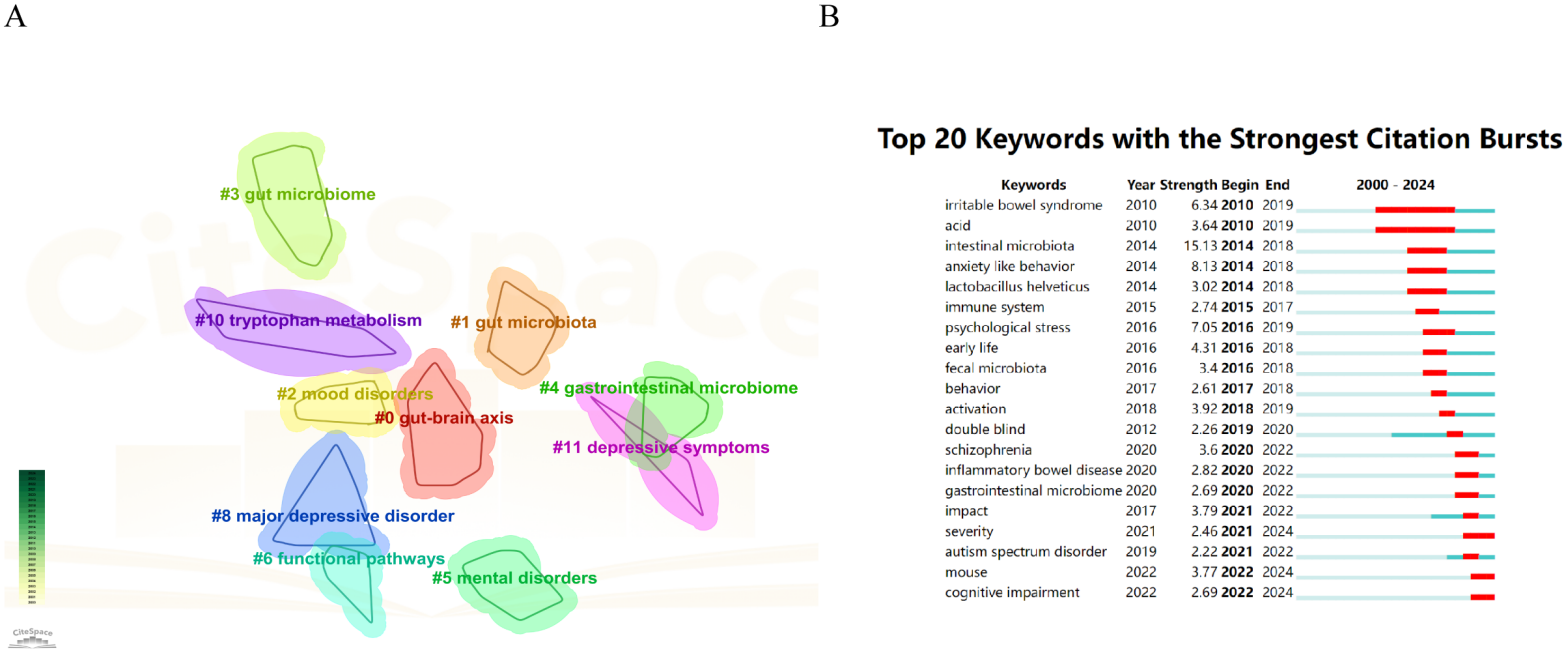
Visualization of keywords related to depression and intestinal flora research. **(A)** The clustered network map of keywords in intestinal microbiota and depression. **(B)** The top 2o keywords with the strongest citation bursts. A blue bar represents the time period in which the keyword appeared; a red bar represents the interval in which the keyword was found to burst, indicating the start year, the end year and the duration of the burst.

**Figure 8A** illustrated the keyword clustering, revealing ten distinct clusters of keywords, including 0# gut-brain axis, 1# gut microbiota, and 2# mood disorders, among others. These clusters represented significant areas of research related to depression and intestinal flora. The primary keywords associated with each cluster were detailed in **Table 6**. These topics not only highlighted the prevailing research hotspots but also suggested potential future research directions, emphasizing the crucial role of the gut-brain axis in the study of depression and gut microbiota. The microbial-gut-brain axis theory, a central concept in this field, explains how gut microbiota interact with the brain via neural, immune and endocrine pathways to modulate mood and behavior. This theory has significantly advanced research into the relationship between gut microbiota and depression, and provides an important framework for understanding the role of gut microbiota in mental health.

**Table 6:** Keywords clustering of intestinal flora in depression diseases.

Rank	LSI	Keywords
0	gut-brain axis	psychiatric disorders; endocrine system; gene-environment; environmental pollutants; gut microbiota; major depressive disorder; gut permeability; mood disorder; fermented foods
1	gut microbiota	major depressive disorder; treatment response; clinical symptom; gut microbiome; animal models; tryptophan metabolism; ventricular hypertrophy; faecalibacterium prausnitzii
2	mood disorders;	gastrointestinal disorders; mental health; mental health disorders; gut microbiome; mental disorders; ischemic stroke; diagnostic model; machine learning
3	gut microbiome	cognitive deficits; weight gain; atypical antipsychotic drugs; gut microbiota; central nervous system; animal models; glucocorticoid receptor; neurodegeneration
4	gastrointestinal microbiome	gut-brain axis; major depressive disorder; systematic review; skin microbiome; gut microbiota; depressive symptoms; chronic pain; anxiety symptoms; skin microbiome
5	mental disorders	brain-gut axis; blood-brain barrier; disease susceptibility; gut-brain axis; emotional processing; mood disorders; environmental epidemiology; disease
6	functional pathways	rectal cancer; medulla; bacteria; gut microbiota; chronic unpredictable mild stress; medulla; bacteria; sleep disturbance
8	major depressive disorder;	gastrointestinal microbiome; methamphetamine abuse; mental disorder; gut microbiome; antibiotic resistance; affective state; lipid metabolism
10	tryptophan metabolism	depression-like behavior; gastrointestinal disorders; parabacteroides distasonis; gut microbiota; eating disorders; cognitive impairment; medicinal plant; gut bacteria
11	depressive symptoms	anxiety symptoms; chronic pain; major depressive disorder; gut-brain axis; fecal microbiota transplantation; short-chain fatty acids; metagenomic sequencing; mental health

The purpose of keyword burst analysis was to identify new concepts that were frequently cited within specific periods, thereby revealing emerging research hotspots or frontiers in a particular field. This analysis enabled researchers to predict future research trends and potential development directions by observing the frequency and timing of keyword occurrences. The results of the keyword emergence analysis were summarized and presented in chronological order, as illustrated in **Figure 8B**. The keyword “intestinal microbiota” emerged as the most prominent, with an intensity of 15.13, and it garnered significant attention from 2010 to 2019. Additionally, “gastrointestinal microbiome” and “autism spectrum disorder” surfaced as notable keywords in this field. The majority of keyword bursts occurred between 2016 and 2022. Furthermore, research on intestinal flora related to depression also peaked in publication during this period.

**Figure 9:**
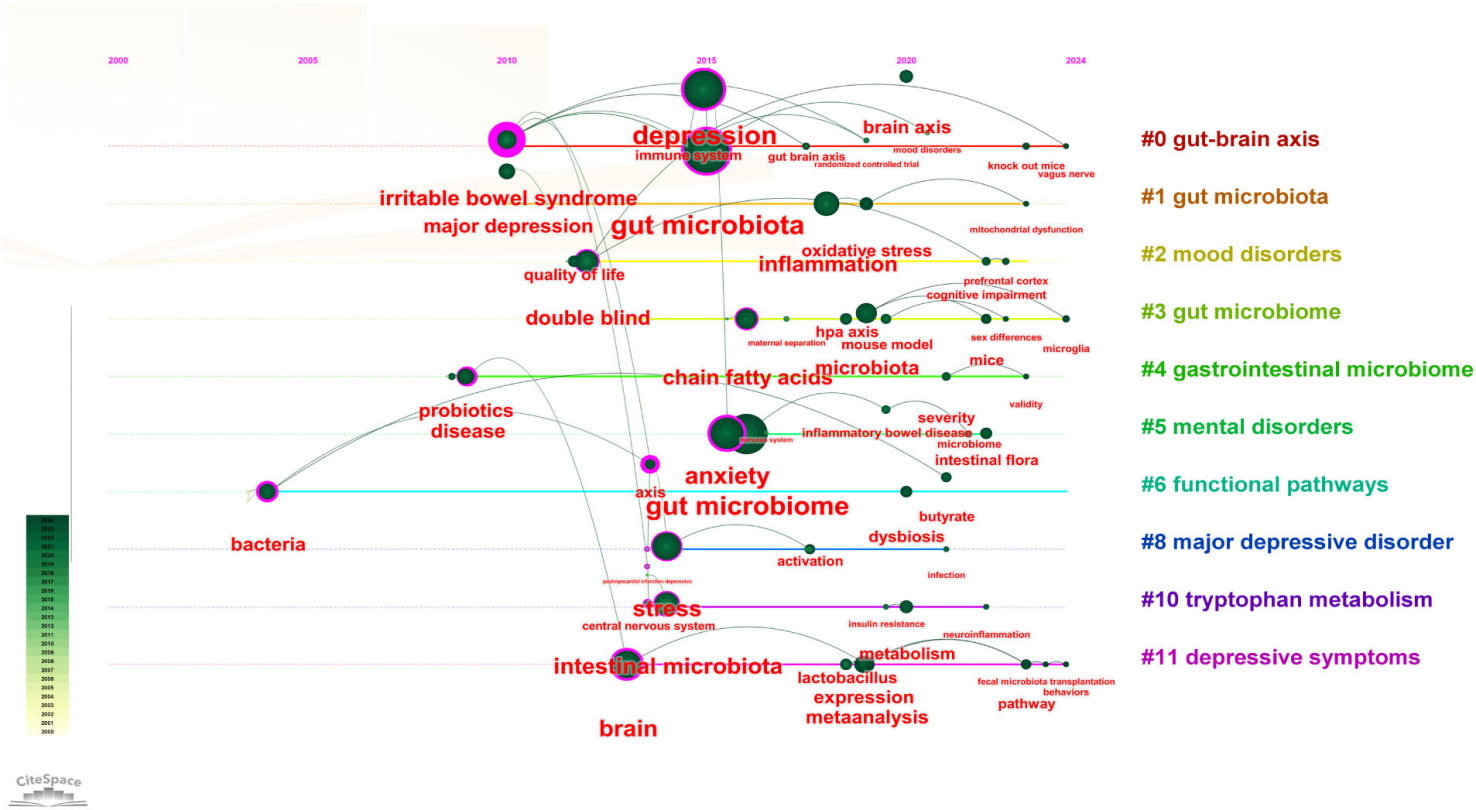
The timeline of clustered network map of keywords in depression and intestinal microbiota.

The timeline graph, as illustrated in **Figure 9**, tracked the fluctuating attention given to specific research topics over time. It enabled researchers to identify emerging trends and predict future research directions. From 2000 to 2015, there was a sparse yet increasing interest in keywords such as “bacteria,” “probiotics,” “Bifidobacterium,” “irritable bowel syndrome,” “depression,” “stress,” and “gut microbiota.” During this period, the incidence of depression gradually increased and became a major global public health problem. However, the limited effectiveness of traditional treatments, such as medication and psychotherapy, has prompted scientists to search for new treatment strategies. Research has focused on descriptive studies and preliminary association analyses, such as looking at differences in gut microbiota between people with depression and healthy people. Keywords such as “bacteria,” “probiotics,” and “gut microbiota” are beginning to emerge, but research depth is limited. In the period from 2016 to 2020, as understanding of the complexity of depression grew, researchers realized the need to explore its pathogenesis more deeply. During this period, the research focus shifted to the in-depth exploration of disease mechanisms, and key words such as “inflammation,” “mechanisms,” “model,” “meta-analysis,” and “brain axis” became research hotspots. In addition, the development of multi-omics technology has allowed scientists to study the relationship between gut microbiota and depression at multiple levels (e.g., genomics, metabolomics). Since 2021, with a deeper understanding of gut-brain axis function, researchers have begun to explore intervention methods based on gut microbiota, and keywords such as “butyrate”, “neurodegenerative diseases”, “tryptophan metabolism” and “pathway” have become research hotspots. For example, studies have identified a potential role for butyrate producing bacteria in improving depressive symptoms. In addition, the role of tryptophan metabolic pathways in depression has also received attention. The long time span of gut-brain axis, functional pathways and depressive symptoms has been the focus of research. Significant advancements have been made, evolving from early observational studies to contemporary molecular biology and neuroimaging research (Ma et al., 2023). The application of new technologies such as artificial intelligence, single-cell sequencing, and spatial transcriptomics offers the possibility of more precisely characterizing the link between gut-brain axis function and depressive symptoms. Future studies are anticipated to further investigate the mechanisms of action of the gut-brain axis in depression, potentially employing more advanced techniques to more accurately delineate the connection between gut-brain axis function and depressive symptoms.

## Discussion

Depression, a universal and complex emotional disorder, poses a significant threat to human health worldwide. The complexity of depression arises from its multifactorial causes, which include genetic predispositions, environmental influences, biochemical imbalances, and psychosocial pressures (Luqman et al., 2024). Given its close association with the nervous system, research has predominantly focused on the biochemical and molecular aspects of its etiology. In recent years, scientific inquiry has increasingly turned to the gut microbiome—a complex ecosystem composed of trillions of microbes that are intricately linked to the health and disease states of their hosts. Emerging evidence suggests that the gut microbiota composition in individuals with depression differs markedly from that of healthy individuals (Góralczyk-Bińkowska et al., 2022; Hao et al., 2020; Mihailovich et al., 2024). Through bibliometric and visual analysis, this study aims to systematically elucidate the role of gut microbiota in depression research, thereby providing a reference for future research directions and clinical applications.

### Overall research characteristics analysis

After screening, a total of 1,046 publications were included in the analytical framework of this study. The steady increase in annual publications indicates that the relationship between depression and gut microbiota has emerged as an active area of research. Advances in technology, increased social demand, and governments and research institutions through research funding, international cooperation programs, and policy support have contributed to the advancement of research in this field (Bremner et al., 2020). At present, research in this domain was predominantly concentrated in Europe and the United States. European and American countries not only have high-level research institutions, but also actively participate in international research networks and cooperation projects, promoting data sharing and resource integration, and further enhancing their status as a link in the international research network. China’s outstanding performance in this field can be attributed to the significant increase in investment in scientific research in China in recent years, strongly supporting research on gut microbiota and mental health. Secondly, China has a large population base and rich clinical resources, which provides a unique advantage for conducting large-scale cohort studies and clinical trials. In addition, Chinese research institutions are increasingly cooperating with top international research teams, further enhancing their academic influence in the field. Given the current trend, further strengthening the ties between research institutions—particularly across different countries and regions—through the formation of multidisciplinary teams could facilitate the integration of expertise from various fields, thereby enabling a more comprehensive exploration of the complex relationship between depression and gut microbiota.

By publishing high-quality research results, journals in related fields reveal research hotspots and frontier trends in the field of depression and intestinal microbiota, providing a core theoretical framework for research in this field, and playing an important role in promoting interdisciplinary cooperation and international academic exchanges and cooperation (Trzeciak and Herbet, 2021). Highly cited journals and literature have played an irreplaceable role in promoting the development of the research field of depression and intestinal microbiota, focusing not only on basic research, but also on the translation and application of research results. In the future, with the advancement of technology and in-depth research, highly cited journals and literature will continue to lead the research hotspots and cutting-edge trends in the field, providing new scientific basis and strategies for the prevention and treatment of depression, and ultimately benefiting patients worldwide.

### Research hot spots and trends

Analysis of keyword co-occurrence, clustering and emerging trends revealed that current research focuses on key areas such as gut microbiota, depression, anxiety, fecal microbiota and the gut-brain axis. Between 2000 and 2015,research in this area remained at the ‘exploratory’ stage, but some studies were beginning to reveal potential links between the gut microbiome and stress and mood, setting the stage for follow-up research (Grau-Del-Valle et al., 2023). Compared to healthy individuals, people with depression had significantly lower gut microbial richness and diversity, and showed an enrichment of pro-inflammatory bacteria and a decrease in anti-inflammatory bacteria (Wang et al., 2023; Yang et al., 2020). High-throughput sequencing techniques and multi-omics analysis tools were not yet widely available, limiting the depth and breadth of research.

Since 2016, there has been a “rapid” increase in research. The increasing social demand, advances inhigh-throughput sequencing technology, metabolomics and neuroimaging technology, the proposal and popularization of the “microbe-gut-brain axis” theory, the promotion of high-impact research, and the cross-integration of microbiology, neuroscience, immunology and psychology have promoted the in-depth development of research. Research has put more emphasis on the mechanisms by which gut microbiota influence depression and microbiome-based interventions (Hu et al., 2016; Zhou et al., 2023; Liu et al., 2019). This shift indicates a transition from preliminary association exploration to a more in-depth investigation of the role of gut microbiota in the pathogenesis of depression. The role of gut microbes and their metabolites in depression presents a double-edged sword (Liang et al., 2018; Liu et al., 2023b), as they not only contribute to the disease’s pathogenesis (Bear et al., 2020) but also offer new perspectives for treatment (Averina et al., 2024; Zhu et al., 2022; Braga et al., 2024; Belelli et al., 2024; Cheng et al., 2024; Gong et al., 2024; Makris et al., 2021; Barbosa et al., 2024).

### Future research directions

At present, the literature in this area is predominantly basic research oriented. Although remarkable progress has been made in the field, there are still many issues that need to be addressed. For example, it remains unclear whether microbial dysbiosis is causal or merely a byproduct of the pathological changes associated with depression (Kumar et al., 2023), and the efficacy and safety of microbiome based interventions in clinical practice still need to be further validated. Future research should focus on mechanism exploration, clinical trial design and the translation and application of research results, in order to provide new scientific basis and strategies for the prevention and treatment of depression. At the same time, strengthening international cooperation and data sharing will help accelerate research progress and clinical applications in this field.

Studies in animal models have mainly explored the mechanism of gut microbiota by simulating the symptoms of depression in humans. Research has tended to delve deeper into the causal relationship between gut microbiota and depression, as well as the effects of specific microbiota or metabolites on depression, providing a theoretical basis for future interventions. For instance, Lactobacillus and Bifidobacterium have been demonstrated to mitigate depression-like behavior in animal models. The proposed mechanisms of action may involve the regulation of the intestinal environment, enhancement of mucosal barrier function, and modulation of neurotransmitter synthesis. Additionally, microbial metabolites, such as short-chain fatty acids and innovative multi-strain E3 probiotics, exhibit significant potential in the treatment of depression (Chan et al., 2023; Alatan et al., 2024; Docampo et al., 2024; Bashir et al., 2024). Beyond probiotics, other microbiota-based interventions, including fecal microbiota transplantation, the use of prebiotics, and Biostime, have also shown promise in altering gut microbiota composition, potentially yielding therapeutic effects for patients with depression (Alli et al., 2022; Chudzik et al., 2021).

Clinical research is directly related to the treatment and health management of patients and therefore has its own unique importance and urgency. Human studies have focused more on specific changes in the gut microbiota of people with depression and explored associations between these changes and depressive symptoms (Radjabzadeh et al., 2022). At the same time, these studies are also more focused on clinical applications, exploring how to improve depressive symptoms by regulating the gut microbiota, including evaluating the efficacy of interventions such as probiotics, fecal microbiota transplantation, and so on (Huang et al., 2016; Lin et al., 2023; Asad et al., 2024).

In general, animal model studies and human studies in the field of gut microbiota and depression have different focuses, with animal model studies contributing to the understanding of disease mechanisms and human studies focusing more on clinical applications and treatment effects. The growth of basic research provides a rich scientific foundation and innovative potential for future clinical applications. The two are complementary and together promote the use of gut flora in the treatment of depression. Taking into account the above analyses, future research trends may include (1) Future studies will focus not only on depression itself, but also on other depression-related disorders or specific physiological processes. (2) The use of new experimental techniques and detection indicators in relevant research to produce more convincing research conclusions. (3) The methods of regulating the gut microbiota will be further refined, with specific mechanisms and applications of how exercise, diet and pharmaceuticals regulate the gut microbiota becoming a focus of research.

## Conclusion

Utilizing scientometric software such as CiteSpace, this study conducts a systematic visual analysis of the literature concerning depression and gut microbiota. By thoroughly examining the current research status, hotspots, and frontier issues in this field, the study aims to provide insights and reference value for future research. Research into the relationship between depression and gut microbiota is advancing rapidly; however, it encounters numerous challenges. Future studies are essential to explore the mechanisms through which the microbiota influences depression and to address the barriers to clinical application. Through interdisciplinary collaboration and innovative research methods, new strategies for the prevention, diagnosis, and treatment of depression are being developed.

### Limitations

In this research, we conducted a comprehensive bibliometric analysis of the literature related to depression and gut microbiota. It is essential to acknowledge several limitations inherent to our study that could be addressed in future investigations. Firstly, our data collection was restricted to the Web of Science Core Collection (WoSCC), which may have excluded relevant literature from other databases. Secondly, the study was limited to English-language publications, thereby neglecting valuable contributions in other languages. Lastly, the selection criteria may have inadvertently overlooked recently published high-quality articles due to their nascent citation profiles.
